# Powdery Mildew Resistance Genes in European Barley Cultivars Registered in the Czech Republic from 2016 to 2020

**DOI:** 10.3390/genes13071274

**Published:** 2022-07-18

**Authors:** Antonín Dreiseitl

**Affiliations:** Department of Integrated Plant Protection, Agrotest Fyto Ltd., Havlíčkova 2787, CZ-767 01 Kroměříž, Czech Republic; dreiseitl@vukrom.cz; Tel.: +420-573-317-139

**Keywords:** *Blumeria graminis* f. sp. *hordei*, *Hordeum vulgare*, pathogen isolates, infection response arrays, resistance gene postulation

## Abstract

Barley is an important crop grown annually on about 55 Mha and intensively cultivated in Europe. In central and north-western Europe, spring and winter barley can be grown in similar environments which creates suitable conditions for the development of barley pathogens, including *Blumeria graminis* f. sp. *hordei*, the causal agent of powdery mildew. Apart from pesticide application, it can be controlled by inexpensive and environmentally-friendly genetic resistance. In this contribution, results of the resistance gene identification in 58 barley cultivars to powdery mildew are presented. In 56 of them their resistances were postulated and in two hybrid cultivars a recently developed method of gene identification was used. In total, 18 known resistance genes were found and several unknown genes were detected. In spring barley, a gene of durable resistance *mlo* is still predominant. *MlVe* found in winter SU Celly was the only new resistance gene recorded in barley cultivars registered in the Czech Republic in this time span. Since 2001 eight new genes of specific resistance have been identified in cultivars registered in the country and their response under field conditions is discussed, including the corresponding responses of the pathogen population due to directional selection. Different strategies for breeding spring and winter barley are recommended.

## 1. Introduction

In Europe barley (*Hordeum vulgare* L.) plays a more important role in crop cultivation than elsewhere in the world. In 2020, 46% of the global area of barley was grown in the subcontinent totaling more than 60% of world production. In the Czech Republic, the crop occupied 332 thousand ha and 1.82 Mt of grain was harvested [1]. In the same year, 124 barley cultivars (68 spring and 56 winter) were registered in the country [2], but the cultivated spring crop was almost at a historical low of only 217 thousand ha [3]. Only 15 cultivars were of domestic origin, but they occupied 50% of the growing area. Winter barley was grown on 115 thousand ha and the only two domestic cultivars comprised 1.2% of the seed propagation area [4].

In the country the ratio of winter to spring barley is almost exactly 1:2. These proportions create good epidemiological conditions for regular infection of cultivars with low resistance to diseases caused by the airborne pathogens [5] including the fungus *Blumeria graminis* (DC.) Golovin ex Speer f. sp. *hordei* Em. Marchal (*Bgh*).

Barley powdery mildew caused by *Bgh* can be controlled by inexpensive and environmentally-friendly genetic resistance or suitable fungicides [6]. Effective resistance protects cultivars and reduces the production of inoculum that could infect other cultivars including those that are susceptible. Many major resistance genes [7,8] and fungicides have been used to control powdery mildew, but numerous agro-chemical compounds [9] and genes of specific resistance have lost their protective effect due to adaptation of the pathogen [10,11].

Adaptive evolution of *Bgh* [12] takes place in large populations that permit frequent mutations from avirulence to virulence [13], mixed reproduction—i.e., alternating vegetative and generative cycles—almost unlimited recombination ability, polycyclic infection during the vegetative growth stages with explosive reproduction, and migration through the long-distance dissemination of airborne spores [14].

In Central and Northwestern Europe, these general characteristics of the pathogen are enhanced by a long-term and massive use of diverse cultivar resistances [15], a high concentration of barley forming an almost perfect “green bridge” optimized by mild climatic conditions that enable joint cultivation of spring and winter barley. During a period of cold, *Bgh* reproduces on its winter host. In the ensuing summer, the vegetation of spring and winter types are linked by a great mass of volunteer plants, which carry the same genes of specific resistance as their parent cultivars, but which often differ between these two forms. All these factors promote directional selection of virulent pathotypes on cultivars with diverse genes of specific resistance and result in a high virulence complexity and pathogen diversity [16]. To obtain sufficient durability of resistance against powdery mildew by breeding cultivars with specific resistances and their combinations is difficult.

The determination of genes of specific resistance against diseases [17,18], later described as ‘postulation’ [19], began to be widely used after the elucidation of the genetic relationships between host-specific resistance and the virulence of a pathogen [20], an interaction subsequently known as the gene-for-gene hypothesis [21], concept [22] or model [23]. The gene postulation of specific resistance against powdery mildew in barley cultivars was carried out mainly in North-western (Denmark, France, the Netherlands, Sweden, United Kingdom) and Central Europe (Austria, Czech Republic, Germany and Poland), and already in 1991 resistance genes of 699 European varieties were collated into a catalogue [15].

The postulation of specific resistances is based on recording the phenotypic responses of a variety after inoculation with pathogen isolates to obtain an infection response array (IRA) also known as the resistance spectrum [24], resistance profile [25] or reaction (response) type array [26,27]. Comparing the IRAs of tested varieties with IRAs of standard genotypes possessing known resistance genes results in the postulation of known or unknown genes and gene combinations.

Resistance to powdery mildew had already been studied in all barley cultivars registered in the country until 2015 [27] and similar studies are extended in this report, which will address the following questions: (i) Do all the tested cultivars contain one or more major resistance genes to powdery mildew? (ii) Are there any new resistances present in the series of cultivars under test? (iii) Does the gene *mlo* of non-specific resistance still play a key role in the resistance of spring cultivars? (iv) What was the effectiveness of resistance genes newly used in this century? and (v) What kind of resistances can be recommended for breeding spring and winter barley?

## 2. Materials and Methods

### 2.1. Plant Material

In the period 2016–2020, a total of 57 commercial barley cultivars were registered and the resistance genes of 55 of them are postulated here. Spring barley Zeppelin registered in 2012 whose resistance was not identified in the previous study [27] was added. Resistance of a hybrid winter cultivar SU Hylona was recorded earlier and another hybrid SY Maliboo was studied here using a new method as described for SU Hylona [28]. For SY Maliboo, 1920 leaf segments were obtained from plants derived from seeds harvested from 40 separate spikes.

### 2.2. Pathogen Isolates

For resistance tests 62 selected reference isolates of *Bgh* were used (for SY Malibo, 48 isolates), which had been collected in 12 countries in all nonpolar continents over a period of 68 years (1953–2021) and comprised the global virulence/avirulence diversity of the pathogen. Before inoculation all isolates were checked for their purity and their correct pathogenicity phenotypes were verified on standard barley lines [29]. The isolates were multiplied on leaf segments of the susceptible cultivar Stirling.

### 2.3. Testing Procedure

About 60 seeds of each accession were sown in two pots (80 mm diameter) filled with a gardening peat substrate and placed in a mildew-proof greenhouse under natural daylight. The primary leaves were excised when the second leaves were emerging, and leaf segments 15 mm long were cut from the middle part of healthy fully expanded leaves. Three segments of each accession were placed on the surface of the media (0.8% water agar containing 40 mg−L of benzimidazole—a leaf senescence inhibitor) in a 150 mm Petri dish. Leaf segments were placed adjacent to each other along with four segments of susceptible Stirling oriented diagonally with their adaxial surfaces facing upward.

For inoculation, a cylindrical metal settling tower of 150 mm diameter and 415 mm in height closed at the top was used, and a dish with segments was placed at the bottom of the tower. Conidia of each isolate taken from a leaf segment of the susceptible cultivar with fully-developed pathogen colonies were shaken onto a square piece of black paper (40 × 40 mm) to visually control the amount of inoculum deposited. Then, the paper was rolled to form a blowpipe and conidia of the isolate were blown through a side hole of 13 mm diameter with its center 50 mm from the upper end into the settling tower over the Petri dish at a concentration of ca. 10 conidia mm^−2^. The dishes with inoculated leaf segments were incubated at 20 ± 1 °C under cool-white fluorescent lamps providing 12 h light at 30 ± 5 μmol m^−2^ s^−1^.

### 2.4. Evaluation

Seven days after inoculation, infection responses (IR = phenotype of accession × isolate interaction) were scored on a scale of 0–4 [30], where 0 = no mycelium and sporulation, and 4 = strong mycelial growth and sporulation. IRs 3, 3–4 and 4 were considered susceptible. Each accession was tested with a minimum of two replications. If there were significant differences in IRs between replicates, additional tests were done. A set of 62 IRs provided an infection response array (IRA) for each accession. Based on the gene-for-gene hypothesis [22], the resistance genes in accessions were postulated by comparing their IRAs with previously determined IRAs of standard barley genotypes possessing known resistance genes. During phenotyping special attention was paid to boundary IRs 2–3 and 3 separating resistance and susceptibility, which pose the greatest risk of error in distinguishing between resistance and susceptibility [31]. Other details of Material and Methods have been recently described [32].

## 3. Results

In 2016–2020, 57 barley cultivars were registered in the Czech Republic. Resistance of Zeppelin could not be identified in a previous study [27] and was added to this set. Therefore, in this paper, the resistance of 56 cultivars was postulated. Hybrid cultivars SU Hylona and SY Maliboo have been studied using a different method [28], the former within this cited article. Hence, the results of resistance gene determination in 58 barley cultivars to powdery mildew are presented herein.

Nineteen IRAs were recorded among postulated cultivars and indicated 18 known resistance genes. However, only three of these were found singly in the tested cultivars, while 15 genes were combined with other genes. Therefore, the set of IRAs was supplemented by 15 IRAs characterizing the phenotype of these genes. A fully susceptible IRA demonstrating the absence of any major resistance gene was also added. Twelve isolates were sufficient to characterize the 35 IRAs (Table 1).

Among 29 cultivars of spring barley, seven known resistance genes were found. In 21 cultivars, the durable resistance gene *mlo* with a characteristic phenotype IR0(3) and effective against all isolates used was present. This gene was also detected in one of the two lines of Spitfire, while the other line contained a combination of three *Ml* genes *a1*, *g* and *La*. The other two cultivars, Adam and Leenke, were also resistant to all the isolates used. However, in both cases in addition to rare IR0(3), IRs of hypersensitive reactions typical for specific resistances were recorded—often IR2 in Adam and less frequently IR1 in Leenke. Therefore, it seems likely that both of these cultivars have an unknown specific resistance, although the presence of *mlo* cannot be excluded, especially in Leenke. Bente, Clarinet, Pop, Remark and Zeppelin contained *MlSI-1* and Pionier *MlRo* in combination with *Mla8*.

Tests of six two-rowed winter barleys revealed five resistance genes, none of which was identical to the seven genes recorded here in spring cultivars. Valerie contained *Mla7*, which is more typically present in spring barleys. In other cultivars, *Mlra* and *Mlh*, found almost exclusively in winter barley, and *MlCh*, known to be in both growth types, were revealed. SU Celly carries *MlVe* combined with another unidentified resistance.

The greatest genetic diversity of resistance genes was observed in a set of six-rowed winter cultivars, in which there were 11 *Ml* genes, and six of these (*a6*, *IM9*, *aLo*, *aLv*, *Ru2* and *p*) were absent in the cultivars of the previous two sets. Combinations of four identical genes (*a6*, *aLo*, *IM9* and *ra*) were found in four cultivars. In this set there were also two hybrid cultivars, SU Hylona and SY Maliboo. A full set of IRs that developed on leaf segments of SY Maliboo after inoculation with 48 isolates is shown in Table S1. *MlaLo* (RT0 after inoculation with Race I and J-462) and an unknown resistance (RT2 after inoculation with pathotypes GH, C-512 and Z-6) were detected in all tested SY Maliboo plants. This result indicates that both these genes were contained in both parents of this cultivar whereas *Mlh* was present in about half of the tested F_2_ plants and probably originates from one parent. An unknown resistance was found in eight out of the set of 58 cultivars (Table 2).

## 4. Discussion

### 4.1. Spring Barley

The most frequently identified resistance gene was *mlo*, present in at least 22 spring barley cultivars. The first commercial cultivar with this gene was the Dutch Atem registered in 1979 [33], and the first cultivar registered in the Czech Republic was the domestically-bred Forum, registered in 1993 [34,35]. The recessive gene *mlo* represents non-specific resistance; virulence against it has not been found and the possibility of its emergence is not expected. Its effectiveness is highly stable even after 43 years of its presence in commercial cultivars, and despite these cultivars having been predominant in Europe at least in the last three decades. The phenotype of cultivars containing this gene is represented by IR0(3), i.e., the presence of a small number (ca. 1% compared to the susceptible control) of less developed colonies. However, almost all cultivars containing *mlo* carry also one or more specific resistance genes, which, after inoculation with avirulent pathotypes, overlap its characteristic phenotype. Such cultivars are then often characterized by IR0, i.e., a frequent phenotype of new, fully effective but non-durable specific resistances, from which the cultivars based on the durable resistance Mlo are difficult to distinguish. Table 1 shows the wild phenotype (IRA) of this gene.

The presence of *mlo* cannot be excluded from Adam and Leenke. For Adam, the pedigree, which would have been beneficial, was not provided by the breeding company. Leenke was selected from an identical cross as Forman and has in its pedigree Salome—one of the first cultivars carrying *mlo* [33]. Unlike Leenke, in Forman there are no doubts about the presence of *mlo*. Spitfire also contains *mlo*, although only in one of two lines. Thus, this gene is present in 22 to 24 of the 29 spring cultivars.

In Pionier *MlRo* was identified, designated accordingly Roxana—the first known commercial cultivar containing this gene and registered in Germany in 2000. *MlRo* was recorded in Kangoo, the first cultivar with this gene registered in the Czech Republic (2008) and one of whose parents was Roxana. *MlRo* was also found in Marnie [36], one of the parents of Pionier and many other cultivars with this gene. The first five cultivars containing *MlRo* were registered in the Czech Republic in 2008–2010. Because of the probable migration from neighboring countries, especially Germany, and the intensive directional selection, the frequency of virulences to this gene in the domestic population of the pathogen increased from 3.5% in 2009 [37] to 81.9% in 2015 [16].

Four cultivars (Bente, Klarinette, Pop and Remark) were postulated with the resistance SI-1. The same resistance was revealed also in the Danish cultivar Zeppelin, the first with SI-1 cultivated in the Czech Republic. However, the resistance of Zeppelin remained unknown for many years [16,27] and has only been identified in this work. The presence of a putative second gene in Zeppelin (*Mlg*) [27] seems less likely. Zeppelin is a parent of the cultivars containing SI-1 except Pop. The first isolate virulent to *MlSI-1* in the Danish spring cultivar Camilla—different from Austrian winter barley Camilla tested here—was found in an airborne population of the pathogen in 2015 [16] and since then the frequency of this virulence has been gradually increasing.

### 4.2. Winter Barley

In Beckenbauer, two genes, *MlIM9* and *Mla6*, were detected. In the Czech Republic, the first cultivar containing *MlIM9* was the German-bred cultivar Carola, registered here in 2001; it also contains *Mla6* and *Mlra* [38]. After the discovery of *MlLo* in Lomerit and other cultivars [39], this gene was also found in Carola. *MlLo* was later mapped to the *Mla* locus or closely linked to it [28]. Carola is in the pedigree of nine cultivars registered in the Czech Republic, five of which contain *MlIM9* [27], but none of them is part of this set. All four *Ml* genes in Carola (*IM9*, *a6*, *aLo* and *ra*) were subsequently found in several other cultivars including KWS Meridian [26] and its four daughter cultivars Journey, KWS Higgins, Novira and Rumcajs investigated here. However, in 2004, 14.1% of 262 isolates were virulent on Carola [40] and this proportion of isolates virulent to *MlIM9* increased rapidly [41].

*MlLv* was identified in five winter barley cultivars, including the hybrid SU Hylona, which was named as the German cultivar Laverda and registered in the Czech Republic in 2007 [42]. This gene was subsequently located at the *Mla* locus [28]. The phenotype (IRA) corresponding to the presence of *MlaLv* was exhibited in Azrah and in three cultivars (Belissa, LG Zoro and SU Lauvira) this gene is present together with *MlaLo*. However, tests with four selected isolates (Table 3) showed that all five cultivars (four postulated here and the standard Laverda) differed not only in the presence of *MlaLo* but also other unknown genes. In addition, *Mla6* and *Mlra*, which often occur in six-rowed winter barley cultivars, are likely to be in some of these cultivars. Nevertheless, these two genes cannot be detected in the presence of *MlaLv* as the pathogen gene bank does not contain any isolate virulent to *MlaLv* and *Mlra* but avirulent to *Mla6*. If *Mla6*, together with *Mla14* closely linked to *Mla6*, is detected in at least one of these cultivars, it would contain a cluster of four genes located at or near the *Mla* locus (*a6*, *a14*, *aLo* and *aLv*). Cultivars carrying *MlaLv* followed a similar path to the resistance of *MlRo* when the frequency of virulence to *MlaLv* increased from 0.0% in 2008 [43] to 50.5% in 2013 [44].

Jakubus and SU Ellen contained *Mlp* and other genes. The parent of SU Ellen is the German-bred Saturn, which was the first cultivar with this gene registered in the Czech Republic in 2012 and Jakubus is a descendant of SU Ellen. Between 2000 and 2011, more than 800 isolates derived from the domestic airborne pathogen population were tested on *Mlp*-bearing cultivars and none of them was virulent to this gene. The first virulent isolates were detected in the country in 2009 [44], but their low frequency (around 1%) was stable at least until 2017 [16]. However, in 2019, the virulence frequency to this gene increased to approximately 5%, and in 2021 had already exceeded 50% [45]. This sudden change must have been caused by an influx of inoculum from other European countries [46] where cultivars with *Mlp* were cultivated in earlier years (at least in Germany and Poland) and supported by directional selection on cultivars grown here.

The two-rowed barley SU Celly is the first cultivar registered in the Czech Republic in which *MlVe* was found (in addition to an unknown resistance). The gene was designated according to the German naming of Venezia [47], which had been included in the Czech registration trials in 2004. Since 2009, Venezia has been added to the differentiation set for the study of domestic airborne populations and the first virulence was found in 2011 after studying 451 isolates [47]. In contrast to the Czech Republic, cultivars with Ve resistance were grown in Germany, where the pathogen population apparently adapted rapidly to this specific resistance. As a result of spore migration the frequency of a corresponding virulence reached 8.3% in the domestic population in 2017. The registration of SU Celly containing *MlVe* in 2020 came too late for this gene to have any positive effect on its resistance in the field. In spite of that, *MlVe* is the only new resistance gene present in barley cultivars registered in the Czech Republic in the given period.

SU Hylona and SY Maliboo are hybrid winter cultivars. The first hybrid registered in the Czech Republic in 2013 was the Swiss cultivar Hobbit. The identification of resistance genes of hybrid cultivars is usually too difficult for gene postulation, not only because hybrid cultivars (F_1_ generation) are characterized by the higher complexity of resistance genes due to the joint presence of alleles located at the same loci, but also because of the high heterogeneity resulting from multiplication of hybrid seed. Therefore, a method combining gene postulation with classical genetic analysis has been developed [28]. SU Hylona served as one of the six model cultivars for the development of this method. In the current work only one hybrid cultivar—SY Maliboo—was studied. The IRAs of both hybrid cultivars are not listed in Table 1 as they can only be assembled artificially, and as a rule, none of the tested sets of leaf segments derived from plants of F_2_ generation shows the relevant IRAs. Resistance of winter barley cultivars in the field is generally low and the hybrid cultivars studied so far have not led to progress towards greater or more durable resistance [28].

### 4.3. Other Remarks

In Table 2, country of origin of the studied cultivars is included. However, this information is of limited importance in the current conditions of breeding concentration. An example is the historically most successful domestic breeding station in Hrubčice, where three studied cultivars (LG Aurus, LG Ester and LG Monus) were bred. However, their country of origin is France, the country of their owner (Limagrain). Also the “national roots” (the initial germplasm of the cultivars) are weakening as a result of the internationalization of breeding. While 10 cultivars from Hrubčice registered in 1976–1985 were bred from 39 parent components, of which 23 (57.5%) were domestic [35], for 10 cultivars bred in 2005–2021 [27,48] including LG Stamgast, the share of domestic parent components was only 35% (7 out of 20).

The number of genes of specific resistance present in cultivars is increasing; in European cultivars registered in the Czech Republic this has risen by eight since 2001 (including *MlaLo* and *MlLu*—the last was not detected here). Thus, pathogen gene banks cannot keep such a diverse range of isolates for postulation studies to determine the main resistances as well as all possible gene combinations. This is particularly the case for genes for which there are fewer virulent isolates available. The abundance (frequency) of the pathotypes in the field and atmosphere then depends on the suitability of the conditions for allowing evolutionary forces to operate, especially regarding directional selection and migration [12]. The examples discussed in the previous paragraphs comprise the main resistance genes and their impact on a population. As the number of isolates required for more detailed postulation increases, it is possible to focus on the identification of more complex gene combinations as in the case of Saturn [46], or cultivars carrying *MlaLv* (Table 3). Sets of isolates can also be selected and after obtaining the required information can be discarded.

Responses of the pathogen population on cultivars containing six specific resistance genes were described above. One of the most successful adaptations of *Bgh* to a specific resistance due to directional selection and migration was the virulence to *Mla13* [49] and its subsequent breakdown [50]. This feature could be summarized as “from a single mildew colony to European barley epidemics” [8]. It illustrates the ‘bust’ part of the ‘boom and bust cycle’ of specific resistances [12] on a continental scale.

Charles Darwin discussed the role of natural selection in evolution. Directional selection of virulent pathotypes on cultivars with specific resistances is a part of this process which can operate rapidly especially in populations of microorganisms. This corresponds to *Bgh* adapting to specific resistance genes through increased virulence frequencies and results in quickly overcoming the resistances as shown in several examples.

## 5. Conclusions

The monitored set of cultivars confirms the increasing differences in the resistance of spring and winter barley. The resistance of spring cultivars is based primarily on the durable non-specific resistance Mlo. The proportion of newly registered cultivars with this gene increased from 62.9% in 2006–2010 to 75.7% in 2011–2015 [27] and reached 75.9–82.8% during 2016–2020. This is a high proportion for one resistance, but there are no known reasons why the predominant use of this effective resistance should change. In contrast, all winter barley cultivars studied here are characterized by a wide range of specific resistances often with three or more genes, which, however, have only a slight positive effect on their resistance in the field. The longer growing season of winter barley, especially in the mild winters that have prevailed in recent decades, allows the pathogen to adapt faster to non-durable resistances. It is therefore necessary to move away from the use of major genes of specific resistance when breeding new, especially winter, cultivars.

As already stated [8], pyramiding of quantitative genes of nonspecific resistance [51,52] for reducing powdery mildew infection in the field [53,54,55] or using introgressions derived from bulbous barley grass (*Hordeum bulbosum*) [56,57] are promising ways for breeding future winter barleys. The utilization of a wide spectrum of resistances derived from more distant species [58], formerly known as non-host [59], can also be adopted once practical methods have been developed.

## Figures and Tables

**Table 1:** Infection response arrays (IRAs) produced by 12 *Blumeria graminis* f. sp. *hordei* isolates on 35 barley genotypes carrying known *Ml* resistance genes.

*Ml* Resistance					Isolate							
Gene(s)	JAP ^1^	ISR	ISR	DK	CZ	CZ	CZ	CZ	CZ	CZ	CZ	CZ
	R-1	J-462	Y-69	EA30	I-162	I-20	X-30	Y-4	O-11	M-8	X-1	X-8
	1953 ^2^	1979	1979	1986	2009	2011	2012	2013	2016	2017	2021	2021
none	4 ^3^	4	4	4	4	4	4	4	4	4	4	4
*a1* ^4^	4	4	4	0	4	0	4	4	4	0	4	4
*a1*, *g*, *La*	0	4	4	0	4	0	2–3	4	4	0	4	4
*a6*	0	4	4	4	0	4	4	4	4	4	4	4
*a6*, *aLo*, *IM9*, *ra*	0	0	4	0	0	4	2	4	2	4	4	4
*a6*, *aLo*, *p*, *ra*	0	0	4	0	0	2	4	2	2	2	4	4
*a6*, *aLo*, *ra*	0	0	4	0	0	4	4	4	4	4	4	4
*a6*, *aLo*, *ra*, *Ru2*	0	0	4	0	0	4	4	4	2–3	4	4	4
*a6*, *IM9*	0	2	4	2	0	4	2	4	2	4	4	4
*a6*, *ra*	0	4	4	0	0	4	4	4	4	4	4	4
*a7*	0	0	0	1–2	4	4	4	4	4	4	4	4
*a8*	0	4	4	4	4	4	4	4	4	4	4	4
*a8*, *h*	0	4	4	4	1–2	4	4	4	4	4	4	4
*a8*, *h*, *Ru2*	1	4	4	4	1–2	4	4	4	2–3	4	4	4
*aLo*	0	0	4	4	4	4	4	4	4	4	4	4
*aLo*, *Lv*	0	0	4	1	1	1	1	4	1	1	4	1
*aLo*, *ra*	0	0	4	0	4	4	4	4	4	4	4	4
*Ch*	2	4	4	4	4	4	4	4	4	4	4	4
*Ch*, *h*, *ra*	2	4	4	0	1–2	4	4	4	4	4	4	4
*Ch*, *ra*	2	4	4	0	4	4	4	4	4	4	4	4
*g*	0	4	4	0	4	0	4	4	4	4	4	4
*h*	4	4	4	4	1–2	4	4	4	4	4	4	4
*h*, *ra*	4	4	4	0	1–2	4	4	4	4	4	4	4
*IM9*	2	2	4	2	2	4	2	4	2	4	4	4
*La*	4	4	4	2–3	4	4	2–3	4	4	4	4	4
*Lv*	1	1–2	4	1	1	1	1	4	1	1	4	1
*mlo* ^5^	0(3)	0(3)	0(3)	0(3)	0(3)	0(3)	0(3)	0(3)	0(3)	0(3)	0(3)	0(3)
*p*	2	4	4	2	2	2	4	2	2	2	4	4
*ra*	4	4	4	0	4	4	4	4	4	4	4	4
*Ro*	4	4	0–1	0	0–1	4	0–1	0–1	4	4	0	0–1
*Ro*, *a8*	0	4	0–1	0	0–1	4	0–1	0–1	4	4	0	0–1
*Ru2*	4	4	4	4	4	4	4	4	2–3	4	4	4
*SI-1*	0	1	1	0	0	0	0	0	0	0	0	4
*Ve*	0	0	4	0	0	0	4	0	0	0	4	0
*Ve*, *u*^6^	0	0	4	0	0	0	0	0	0	0	4	0

^1^ Country of isolate origin: JAP—Japan, ISR—Israel, DK—Denmark, CZ—Czech. ^2^ Year of isolate collection. ^3^ According to Torp et al. [30], IR4 represents susceptibility. ^4^ Highlighted IRAs were found only in standard lines. ^5^ Wild phenotype. ^6^ u = Unknown.

**Table 2:** Powdery mildew resistance genes in 57 European barley cultivars registered in the Czech Republic from 2016 to 2020 and Zeppelin.

Cultivar	Original	Pedigree	Country	Year of	*Ml* Gene(s)
	Designation		of Origin	Registration	
***Spring*, *Two-Rowed***					
Accordine	AC 10/734/33	(Sunshine × SY Firkin) × SY Firkin	Germany	2018	*mlo*
Adam	NORD 15/1107	NA	Germany	2020	*u*
Aligator	STRG 774/11	Gundel × S99G153 (Braemar × Roxanna)	Germany	2016	*mlo*
Avus	STRG 687/15	Explorer × Shuffle	Germany	2020	*mlo*
Bente	NORD 13/1114	(Vendela × Zeppelin) × Grace	Germany	2018	*SI-1*
Cosmopolitan	SJ 152037	(KWS Irina × Evergreen) × (Sanette × Paustian)	Denmark	2019	*mlo*
Fandaga	NORD 14/2404	Ginger × Britney	Germany	2020	*mlo*
Forman	NORD 12/2444	(Salome × Livia) × Propino	Germany	2017	*mlo*
Ismena	NORD 14/2403	NA	Germany	2019	*mlo*
Klarinett	SC 101-12A	Zeppelin × Grace	France	2019	*SI-1*
KWS Fantex	KWS 13/207	Sunshine × KWS Irina	Germany	2018	*mlo*
Laureate	SY 412-328	Sanette × Concerto	Germany	2019	*mlo*
Leenke	NORD 12/2531	(Salome × Livia) × Propino	Germany	2017	*u*
LG Aurus	LGBHE3427A	Petrus × Zhana	France	2019	*mlo*
LG Ester	LGBHE3254B	Scrabble × Signum	France	2020	*mlo*
LG Monus	HE-2645	HE 204 × Gladys	France	2017	*mlo*
LG Nabuco	LGBN1315	Cropton × LN0925	France	2018	*mlo*
LG Tosca	LGBN14223-2	RGT Planet × LGBN1469	France	2020	*mlo*
Libuše	NORD 11/2411	NFC 403-135 × Grace	Germany	2016	*mlo*
Manta	AC 07/547/417	(Claire × Quench) × Lilly	Germany	2016	*mlo*
Ovation	LGB12-8317-A	NSL 07-8113-B × Tesla	France	2017	*mlo*
Pilote	SY 413357	Saporis × Melius	Switzerland	2018	*mlo*
Pionier	SC 65/03 NZ 7C	Marnie × Beatrix	France	2016	*a8*, *Ro*
Pop	SC 44801 N2	Calcul × SY Firkin	France	2017	*SI-1*
Remark	AC 09/547/43	Zeppelin × Columbus	Germany	2017	*SI-1*
Runner	NORD 14/2534	NA	Germany	2019	*mlo*
Soulmate	NOS 16111-55	Barabas × Keops	Denmark	2017	*mlo*
Spitfire	SG-S 212	STRG 01/410/41 × Westminster	Czech	2018	*a1*, *g*, *La + mlo*
Tango	LN1147	Jazz × Claire	France	2016	*mlo*
Zeppelin ^1^	SJ 071085	(Scandium × Isabella) × SJ 050623	Denmark	2012	*SI-1*
***Winter, Two-Rowed***					
KWS Donau	KW 2-430	(KWS Liga × KWS Stella) × KW 2-936	Germany	2017	*Ch*, *ra*
Neptun	SJ 128045	Sandra × Matros	Denmark	2019	*Ra*
Sobell	SJ 128113	Augusta × KWS Cassia × Matros	Denmark	2019	*ra*, *u*
SU Celly	NORD 13109/14	(NORD 2930 × Valentina) × California	Germany	2020	*Ve*, *u*
Torpedo	AC 08/290/26	KWS Cassia × Augusta	Germany	2016	*Ch*, *h*, *ra*, *u*
Valerie	Br 11500r6	207-589 × Sandra	Germany	2020	*a7*
***Winter, Six-Rowed***					
Azrah	STRG 432/09	Laverda × (Cornelia × Carola)	Germany	2018	*aLv*
Beckenbauer	BE 2008024004D	Kathleen × BE 2718	Germany	2019	*a6*, *IM9*
Belissa	AC 09/275/22	KWS Meridian × Antonella	Germany	2017	*aLo*, *aLv*, *u*
Camilla	SZD 2213A	Semper × Kathleen	Austria	2019	*h*, *ra*
Falbala	LEU 53120	(ST 2475 × Fridericus) × Amelie	Germany	2020	*a6*, *ra*
Impala	LEU 43408	St. 2474 × Federicus × Meridian	Germany	2018	*a6*, *aLo*, *ra*, *Ru2*
Jakubus	NORD 12119/102	Bella × SU Ellen	Germany	2020	*a6*, *aLo*, *p*, *ra*
Journey	KW 6-451	KWS Meridian × KWS Tonic	Germany	2018	*a6*, *aLo*, *IM9*, *ra*
KWS Higgins	KW 6-331	KW 6-855 × KWS Meridian	Germany	2017	*a6*, *aLo*, *IM9*, *ra*
KWS Wallace	KW 6-1541	KWS Tonic × KW 6-148	Germany	2019	*a8*, *h*
Laurin	NORD 11002/8	Tenor × 08076/86 (Kathleen × Saturn)	Germany	2018	*aLo*, *ra*, *u*
LG Triumph	LGBN13W125-43	Souleyka × KWS Meridian	France	2017	*a6*, *aLo*, *ra*
LG Zoro	LGBB15W003	KWS Meridian × Rafaela	France	2019	*aLo*, *aLv*
Novira	AC 09/278/6	AC 07/142/36 × KWS Meridian	Germany	2017	*a6*, *aLo*, *IM9*, *ra*
Pegasos	LEU 63112	(Ramata × ST2426) × ST2427	Germany	2020	*aLo*, *ra*
Rumcajs	STRG 568/15	Kathleen × KWS Meridian	Czech	2020	*a6*, *aLo*, *IM9*, *ra*
SU Ellen	NORD 08076/133	Kathleen × Saturn	Germany	2017	*a6*, *aLo*, *p*, *ra*
SU Hylona ^2,3^	DEH 13/1807	CMS04LM183L001 × 10HR170D002	Germany	2018	*al*, *aLv*
SU Jule	BE 2008108012	Semper × BE 27090	Germany	2018	*a7*
SU Lauvira	NORD 13078/8	NORD 11118/64 × ((BYDW 55 × Kathleen) ×	Germany	2020	*aLo*, *aLv*, *u*
		(Tenor × Loreley))			
SY Maliboo ^2^	SY 216489	(F1F180 × RE35) × MT0767	Switzerland	2020	*aLo*, *h*, *u*
William	KW 6-437	(LP 6-854 × KWS Meridian) × KWS Tonic	Germany	2020	*Ch*, *h*, *Ru2*

^1^ Resistance of Zeppelin could not be postulated previously [27]. ^2^ Hybrid cultivar. ^3^ Resistance was published in a set of hybrid cultivars [28]. *u* = unknown resistance gene. + indicates presence of different genotypes.

**Table 3:** Infection response arrays produced by four *Blumeria graminis* f. sp. *hordei* isolates on five winter barley cultivars carrying *MlaLv* resistance gene.

Cultivar	*Ml*	ISR ^1^	CZ	CZ	CZ
	Resistance	J-462	Y-4	M-4	A-1
	Gene(s)	1979 ^2^	2013	2015	2015
Azrah	*aLv*	4 ^3^	4	4	4
LG Zoro	*aLv*, *aLo*	0	4	4	4
SU Lauvira	*aLv*, *aLo*, *u*	0	2	4	4
Belissa	*aLv*, *aLo*, *u*	0	4	0–1	4
Laverda	*aLv*, *u*	1–2	4	1–2	4

^1^ Country of isolate origin: ISR—Israel, CZ—Czech. ^2^ Year of isolate collection. ^3^ According to Torp et al. [30], IR4 represents susceptibility.

## Data Availability

All relevant data are presented in the article and Supplementary Table S1.

## Supplementary Materials

The following supporting information can be downloaded at: https://www.mdpi.com/article/10.3390/genes13071274/s1, Table S1. Infection responses (IRs) developed on leaf segments excised from plants of F_2_ generation grown from seed of 40 individually harvested ears of hybrid winter barley cultivar SY Malibo after inoculation with 48 *Blumeria graminis* f. sp. *hordei* isolates.
